# The factor structure of the Strength and Difficulties Questionnaire in a national sample of Swedish adolescents: Comparing 3 and 5-factor models

**DOI:** 10.1371/journal.pone.0265481

**Published:** 2022-03-14

**Authors:** Patrik Karlsson, Peter Larm, Johan Svensson, Jonas Raninen

**Affiliations:** 1 Department of Social Work, Stockholm University, Stockholm, Sweden; 2 The Swedish Council for Information on Alcohol and Other Drugs (CAN), Stockholm, Sweden; 3 Department of Clinical Neuroscience, Karolinska Institutet, Solna, Sweden; 4 Department of Public Health, Stockholm University, Stockholm, Sweden; 5 School of Social Sciences, Unit of Social Work, Södertörn University, Huddinge, Sweden; 6 Centre for Alcohol Policy Research, La Trobe University, Melbourne, Australia; University of Texas at Arlington, UNITED STATES

## Abstract

The Strength and Difficulties Questionnaire (SDQ) is one of the most common screening instruments for emotional and behavioral problems in children and adolescents. Although exploratory factor analyses support the originally proposed 5-factor structure of SDQ as well as a 3-factor model, the evidence from confirmatory factor analyses is more mixed. Some of the difficulties items in SDQ are positively worded and it has been proposed that this leads to method effects, i.e. these items share variance that is due to the method used rather than to a substantive construct. Also, there seems to be minor factors in some subscales. This study tests a series of 3- and 5- factor models pertaining to the factor structure of SDQ, also considering method effects and minor factors. The sample consists of a nationally representative cohort of Swedish adolescents born in 2001 (n = 5549). Results show a relatively better fit of the 5-factor model compared with the 3-factor model although neither of these had a satisfactory fit. Model fit was improved when specifying cross-loadings of the positively worded difficulties items on the prosocial scale as well as minor factors on the hyperactivity scale. Although no model provided a completely satisfactory fit to the data, the results show that the 5-factor model performs better than the 3-factor model and has an acceptable fit. We conclude that for the purposes of epidemiological research, SDQ has acceptable factorial validity, provided that researchers consider method effects and minor factors.

## Introduction

The Strength and Difficulties Questionnaire (SDQ) is a widely used instrument to assess emotional and behavioural problems in children and youth. Originally developed in England by Goodman [1], SDQ has been translated to a large number of languages and is indented to be used for both clinical assessments and epidemiology [2]. SDQ consists of totally 25 items, of which 20 capture difficulties and five strengths. The 20 difficulties items are proposed to measure four different subscales, referred to as “emotional problems”, “conduct problems”, “hyperactivity” and “peer problems” and may be combined into a “total difficulties scale”. “Prosocial” is the name of the strength subscale. SDQ is used both for younger children and for adolescents and may be completed by parents, teachers or by adolescents themselves.

A main benefit of SDQ is that it is relatively short [3]. It only comprises a fifth as many questions as the Child Behaviour Checklist (CBCL) and it also has the obvious benefit of being free of charge [4]. It is conceptually strong in being informed by DSM criteria [5], its subscales correlate strongly with corresponding subscales in CBCL [6] and at least moderately with clinical diagnoses [7]. Its focus on strengths in addition to problems is also considered an attractive feature, as this facilitates a broader assessment of children’s behavior [3, 4]. Thus, SDQ has the potential of being a useful tool in epidemiology and for screening for mental and behavioural problems in general population samples of youth [8].

By means of confirmatory factor analysis (CFA), this study aims to assess the factor structure of self-assessed SDQ in a nationally representative sample of Swedish adolescents aged 15–16 years, testing a range of models proposed in prior research on SDQ. While there are prior Swedish studies on the validity of SDQ [9–14], to the best of our knowledge, this is the first Swedish study to test the factor structure of self-assessed SDQ using CFA based on a nationally representative sample of adolescents. Extant Swedish research on self-assessed SDQ is limited and inconclusive. While the original factor structure has been generally supported in a few studies [12, 13], another study concluded that “it seems reasonable to question the assumed unidimensional nature of some of the SDQ subscales” [14, p. 1300]. While the latter did not use factor analytical techniques but Rasch-modelling to assess each of the original scales, it suggests that there may be some central problems with self-assessed SDQ in Swedish youth.

The internal factor structure is an important concern when assessing a scale’s validity: we need to know that the included items measure the constructs accurately. If not, individual scores on the assumed constructs may not reflect what they are supposed to reflect. Prior international research on the factor structure of SDQ is inconclusive and given SDQ’s prominence in the field, it is imperative to assess how well it measures the theoretical constructs it purports to measure across youth from various cultural backgrounds [15]. Specifically, we test a series of 3- and 5- factor models pertaining to the factor structure of SDQ, also considering method effects (due to positively worded difficulties items) and minor factors in the hyperactivity scale. While some prior work has also considered higher order factors (e.g., [16]), the study is restricted to first-order factors.

### Three or five factors?

A range of studies across different countries broadly supports the 5-factor structure of SDQ [3, 5, 8, 9, 17–23]. A meta-analysis of the parent and teacher reported versions of SDQ in children aged 4–12 concluded that the vast majority of the studies supported the 5-factor structure, with the results being most supportive of parental reports [4].

However, other studies have failed to find support for SDQ’s original 5-factor structure [24–26]. McCrory and Layte [3 p. 882] point out that the results seems to be “more equivocal in their support for the putative factor structure [of SDQ]” once researchers move from exploratory to confirmatory factor analysis. The conduct and peer problem scales have also in some studies been found to have unsatisfactory internal consistency, as measured by Chonbach’s alpha (e.g., [14]), and the correlations between some subscales are in some studies quite large (see e.g., [21, 22]), suggesting that they potentially could be collapsed. Richter et al. [21], in a Norwegian study, showed that the correlation between the conduct and hyperactivity scales was so high that it challenged the proposition that these two scales measure different constructs.

Some studies suggest that SDQ may be better conceptualized as comprising only three factors, including an externalizing and an internalizing dimension as well as a prosocial one [27], but other studies have failed to support this proposition [e.g., 3, 25, 28]. Complicating things further, there is evidence to suggest that for self-assessed SDQ the 3-factor model may provide a better fit in some countries, that the 5-factor model fits better in other countries and that none of these fit well in still other countries [15]. In addition, some research suggests that the subscales may not be unidimensional [14, 24].

### Method effects in SDQ

The sometimes-observed poor fit of SDQ has in the literature repeatedly been discussed with reference to “method effects”. “Method effects” or “common method bias” are well-known challenges to measuring instruments [29]. Brown [30] explains this situation as when additional covariance between observed items is introduced because of how the items are measured. An example is when a construct is measured using both positively and negatively worded items [30] as is the case in three of the four difficulties subscales in SDQ. As DiStefano and Motl [31 p. 441] point out,

The assumption underlying the use of positively and negatively worded items on a survey is that the items are measuring the same content, meaning that different ways of wording a question (e.g., positive or negative) should yield equivalent responses (Marsh, 1996). However, if responses differ due to the direction of the item wording, then the resulting data are likely confounded by a method effect caused by the mechanism used to collect the information. This method effect may not only obstruct the researcher’s view of the content under study (i.e., factorial validity), but, more important, influence interpretations that rely on the accuracy of the data.

Failure to account for method effects may lead to erroneous conclusions regarding the number of dimensions in a theoretical construct. Some dimensions may simply be methodological artefacts [32]. Because five of the difficulties items in SDQ are positively worded (SDQ7 in the conduct scale, SDQ11 & SDQ14 in the peer problem scale, and SDQ21 and SDQ25 in the hyperactivity scale), it has been suggested that method effects in part may account for the modest internal consistency of some of the subscales of SDQ [33].

Different ways of accounting for method biases in CFAs have been proposed. Two common options are the “correlated traits, correlated-methods” (CTCM) and the “correlated, traits, correlated uniquenesses” approaches (see e.g. [31]). CTCM specifies in addition to the theoretical constructs separate method factors for item measured using the same “method”, whereas CTCU models method effects through the specification of correlated error terms for all items sharing the same method [31].

Studies specifying method effects in CFAs of SDQ have typically found improved goodness of fit indices [3, 8, 22, 25, 28, 33, 34]. For example, a large Dutch study applying self-reported SDQ in youth aged 11–17 showed that the fit of the 5-factor model was improved after allowing for cross-loadings of the positively worded difficulties items on the prosocial scale [33]. Such cross-loadings have also been identified in exploratory factor analyses and principal component analyses [5, 20, 27]. Other studies have found improved fit when specifying an independent method factor comprising the positively worded items and the prosocial items in addition to the original factors [3, 8]. While these studies propose that a method effect is due to both the positively worded difficulties items and the prosocial items, method effects have also been estimated by restricting the items to the positively worded difficulties items [22]. To the extent that prosocial items indeed constitute a distinct prosocial construct, there may be reasons to restrict the method effects to the positively worded difficulties items.

### Minor factors in SDQ

Specifying method effects, however, may not be a sufficient remedy for the unsatisfactory fit indices found for the original 5- factor model in some studies. For example, in van de Looij-Jansen et al. [33] the best fit was obtained when the researchers in addition to cross-loadings of the positively worded items also specified correlated error variances between some of the other items (i.e., to capture minor factors within some of the subscales). Ortuño-Sierra et al. [28] found the best overall fit indices across the countries involved when allowing both for method effects and for correlated error terms, and this was the case both for the original 5-factor model and the 3-factor model. Percy et al. [25] found significant improvement in the 5-factor model when also allowing for error covariance between two of the hyperactivity items in addition to correlated error terms for the positively worded items. The relatively best fit was obtained when they in addition to this specified cross-loadings for SDQ3 on conduct problems and for SDQ13 on peer problems. Bøe et al. [35] obtained acceptable fit without specifying method effects but allowing for correlated errors between two sets of items on the hyperactivity scale and for a cross loading of the tantrum item (SDQ5, originally proposed to load on the conduct scale) on the emotional scale.

Although several studies suggest that there may be minor factors on e.g. the hyperactivity scale, they have differed in which parameters that have been freely estimated (i.e., not constrained to zero). As is common in practical CFA applications, the fit of the tested models is often unsatisfactory so researchers tend to consult modification indices (MIs) as a guide for how the model could be improved. The use of MIs in guiding model specification is a debated topic and is generally considered appropriate only if there are a priori reasons of so doing. As pointed out by MacCullum et al, [36, p. 492] such search “is inherently susceptible to capitalization on chance in that idiosyncratic characteristics of the sample may influence the particular modifications that are performed”. Thus, the modifications made may not work in other samples. As it seems, the only somewhat consistently freed parameter is the covariance between SDQ items 2 and 10 on the hyperactivity scale. Thus, there are unresolved issues regarding the factor structure of SDQ and how to best specify the models.

More studies are needed to replicate models proposed in the literature across different samples and using different raters. Several CFAs assessing method effects have focused on younger children and thus relied on e.g. parents as raters [3, 8] and these findings may not generalize to samples of adolescents across various contexts. Using CFA we test a series of models proposing different factor structures of SDQ that has emerged in the literature. We thus follow the advice of MacCullum et al. [36, p. 503] to test “multiple a priori models” rather than to exploratively search for the one that fits the current sample data best.

**Fig 1:**
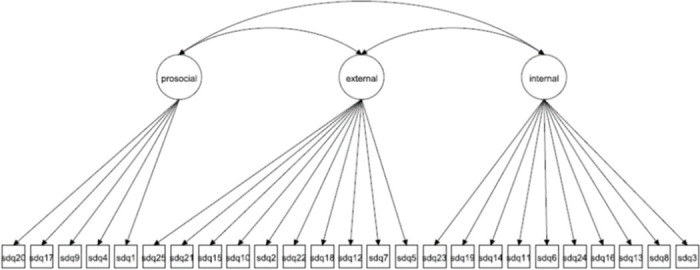
3-factor model (Model 1).

**Fig 2:**
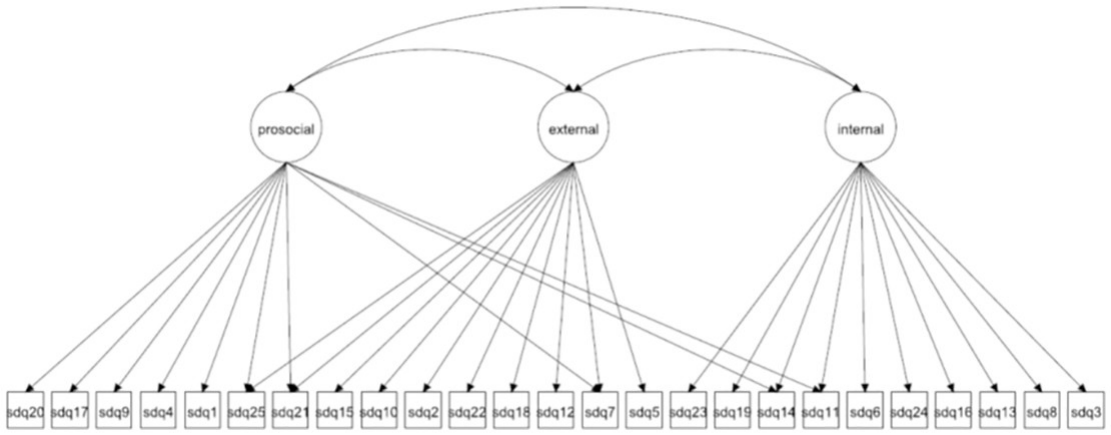
3-factor model with cross-loadings (Model 2a).

**Fig 3:**
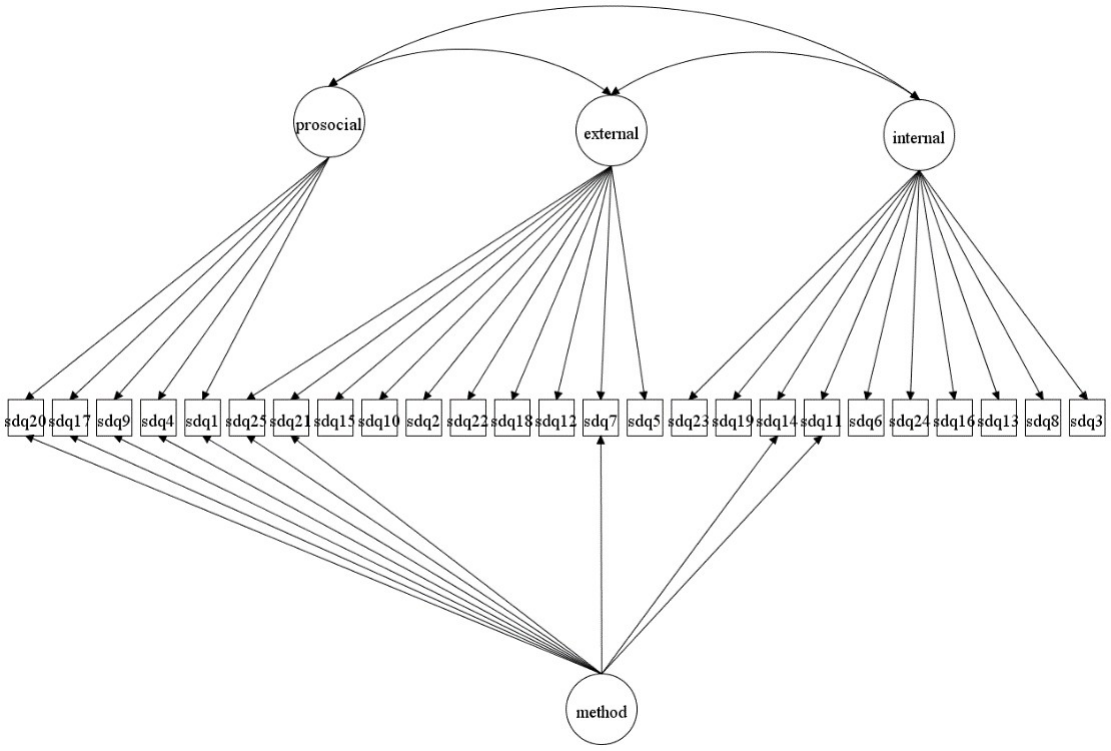
3-factor model with a separate method factor (Model 2b).

**Fig 4:**
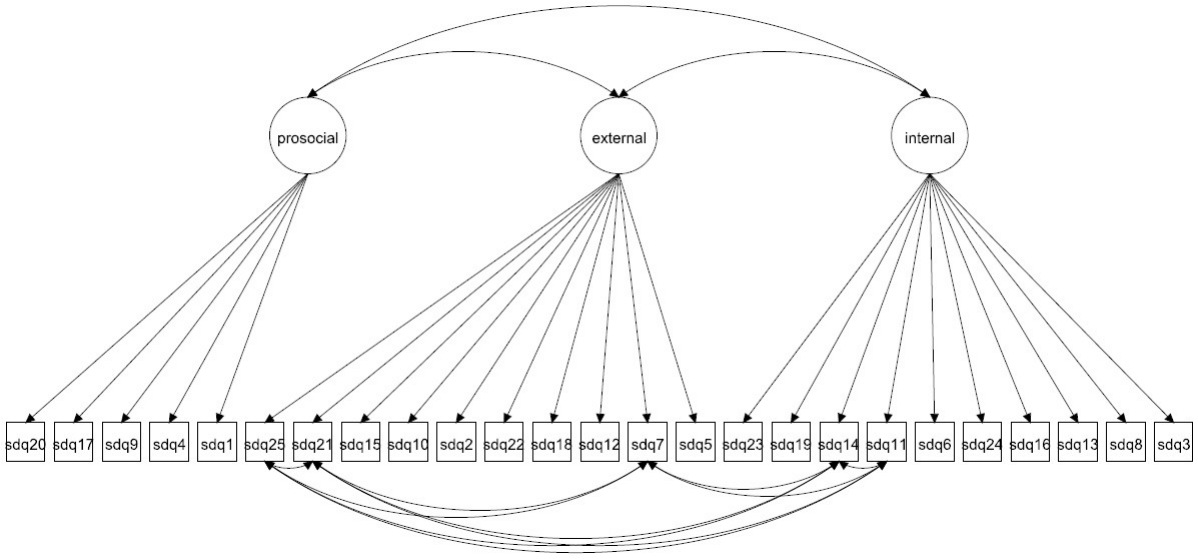
3-factor model with correlated errors for method effects (Model 2c).

**Fig 5:**
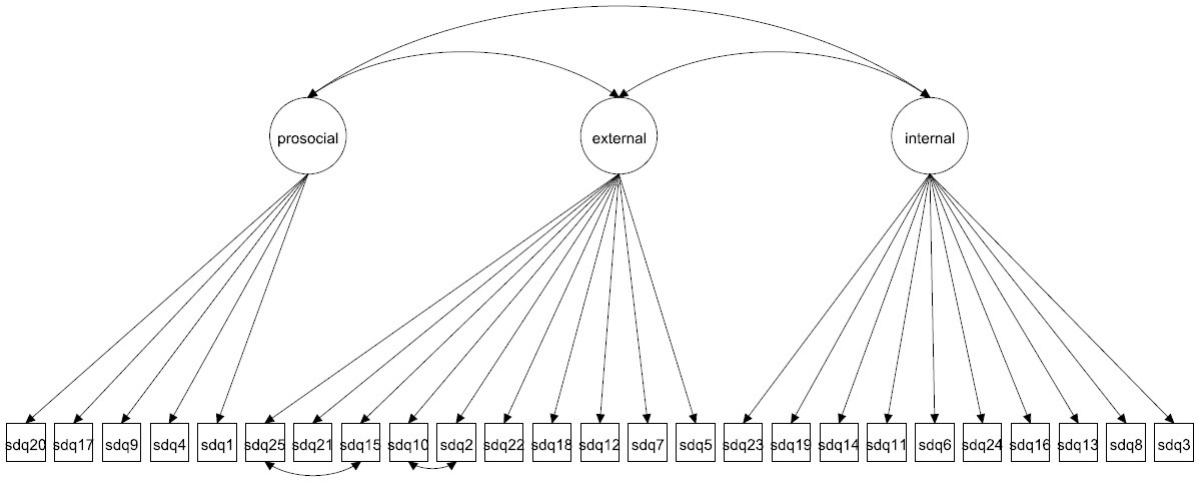
3-factor model with correlated errors for minor factors (Model 3).

**Fig 6:**
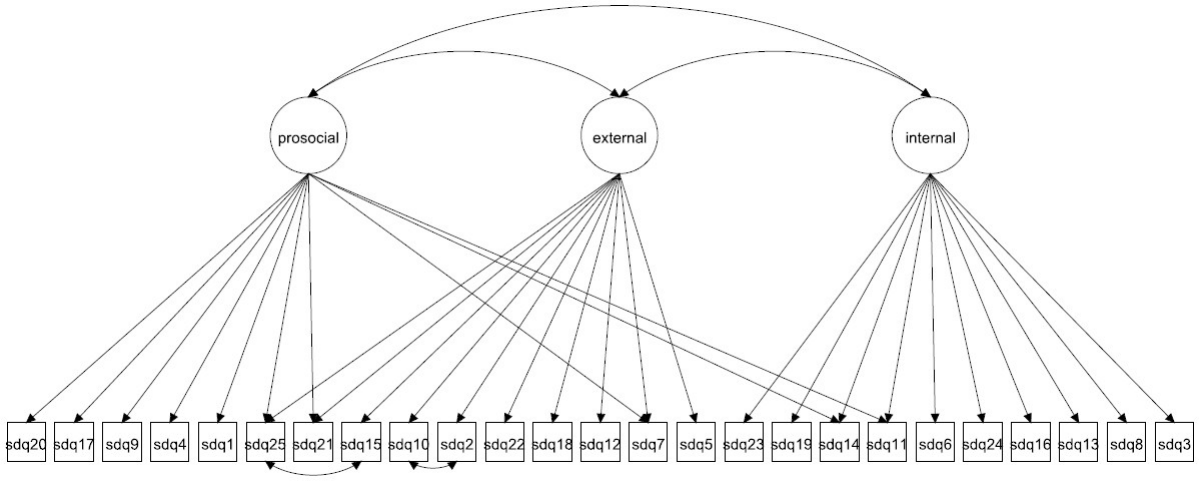
3-factor model with cross-loadings and correlated errors for minor factors (Model 4a).

**Fig 7:**
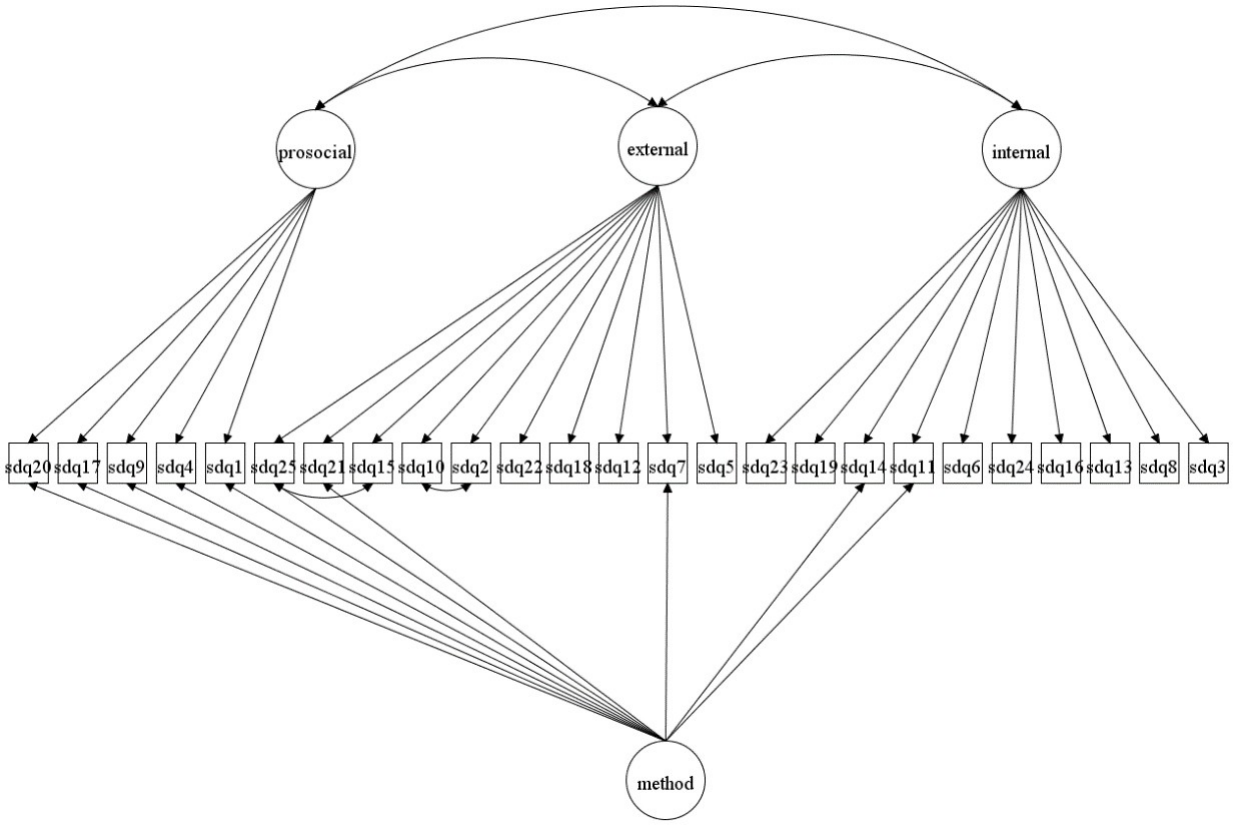
3-factor model with a separate method factor and correlated errors for minor factors (Model 4b).

**Fig 8:**
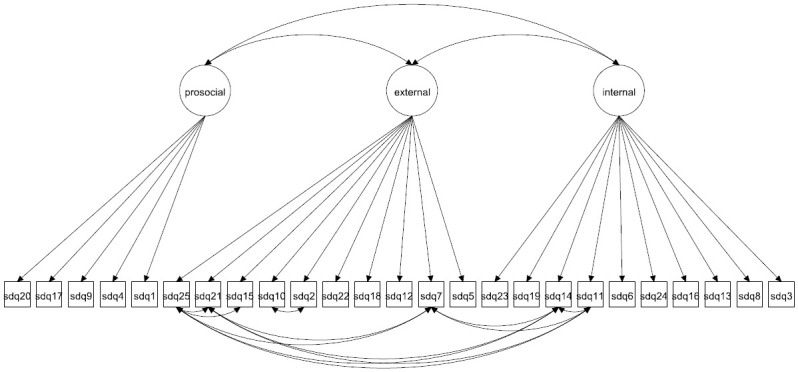
3-factor model with correlated error terms for method effects and minor factors (Model 4c).

**Fig 9:**
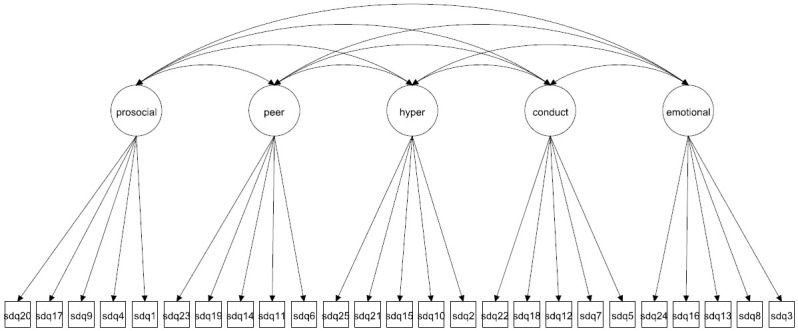
5-factor model (Model 5).

**Fig 10:**
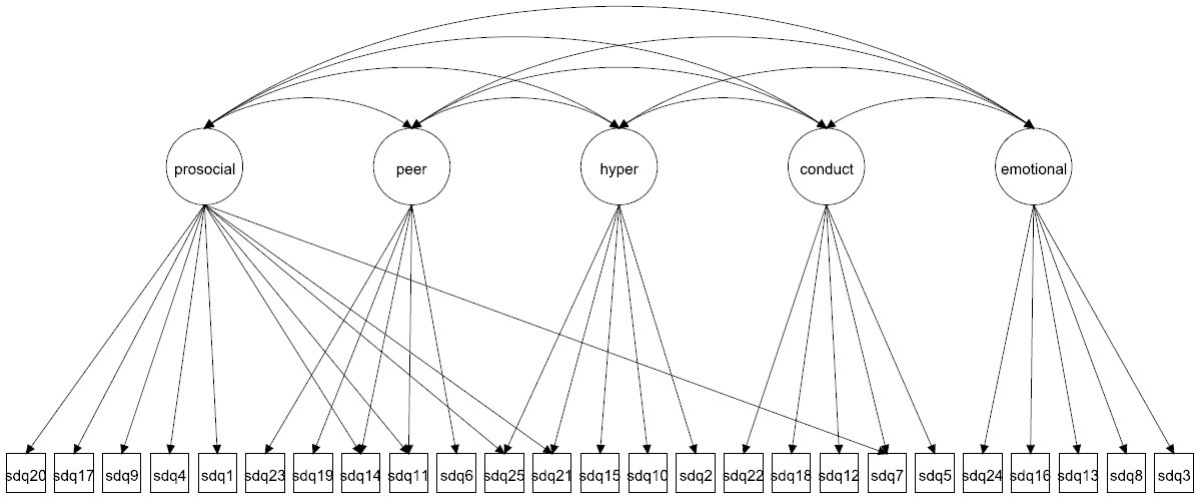
5-factor model with cross-loadings (Model 6a).

**Fig 11:**
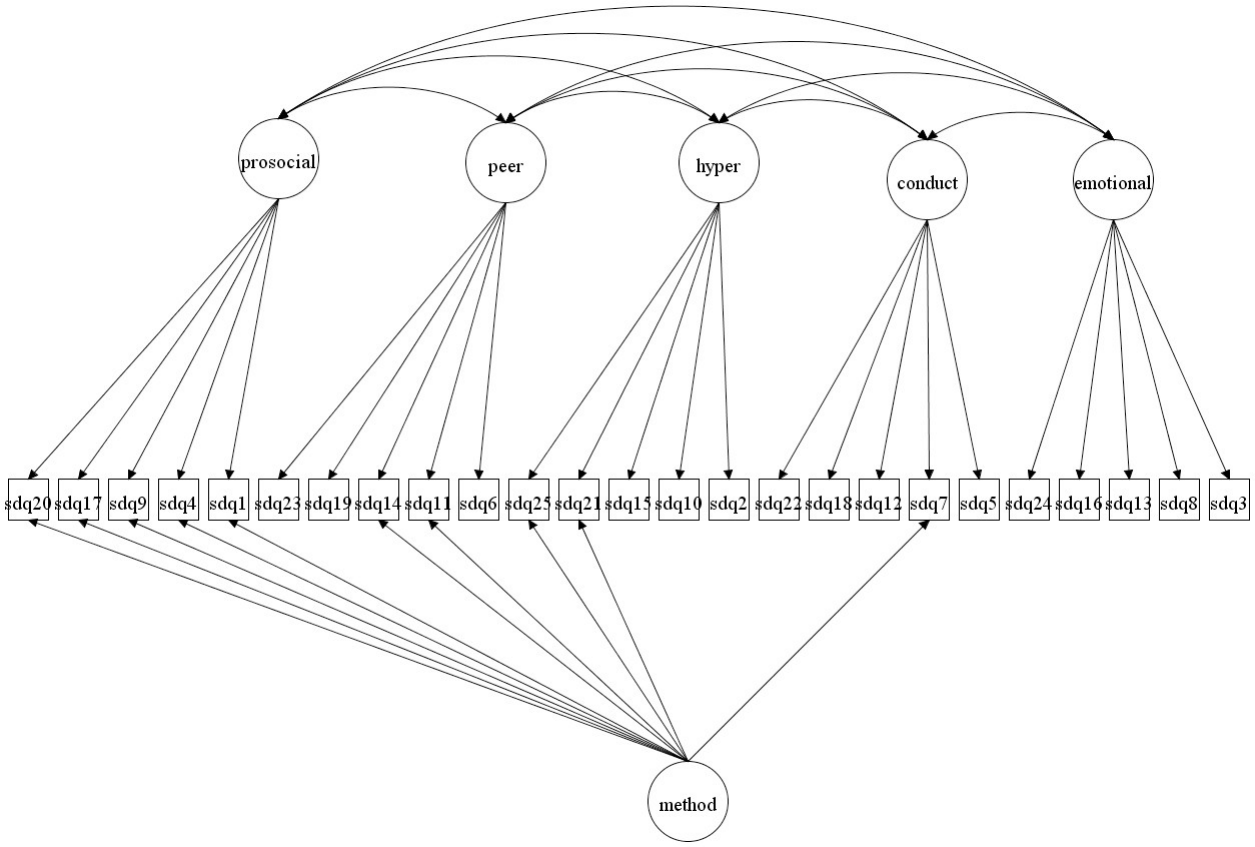
5-factor model with a separate method factor (Model 6b).

**Fig 12:**
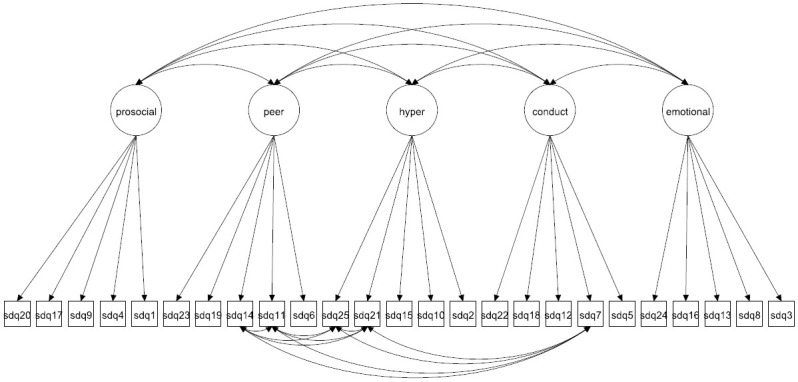
5-factor model with correlated errors for method effects (Model 6c).

**Fig 13:**
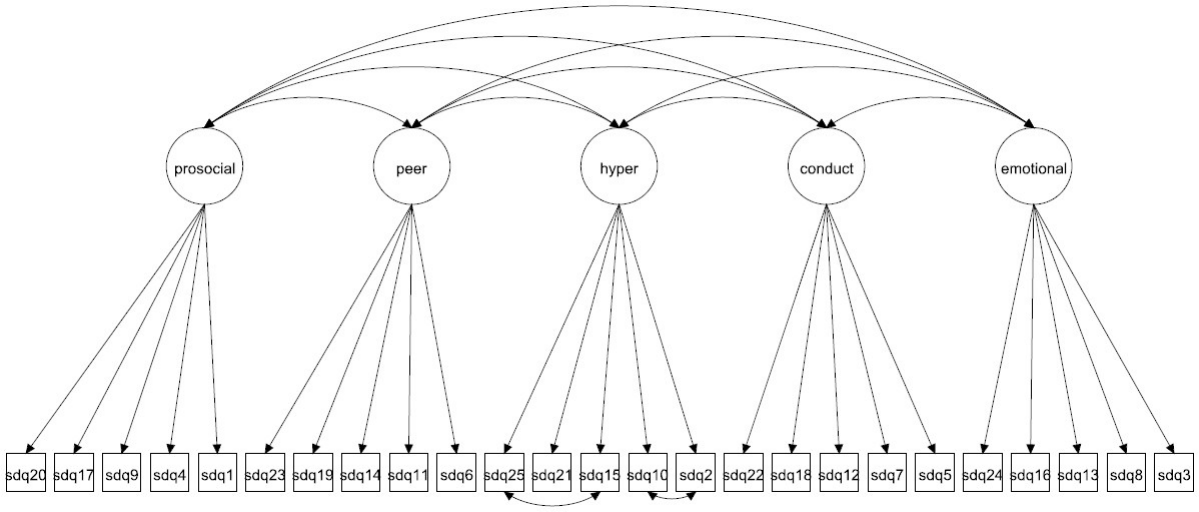
5-factor model with correlated errors for minor factors (Model 7).

**Fig 14:**
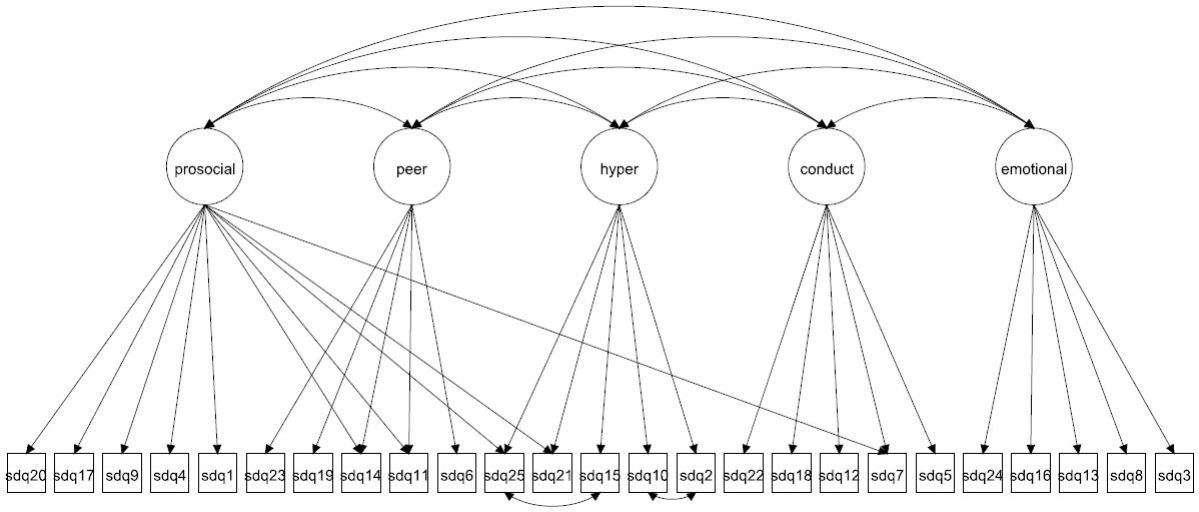
5-factor model with cross-loadings and correlated errors for minor factors (Model 8a).

**Fig 15:**
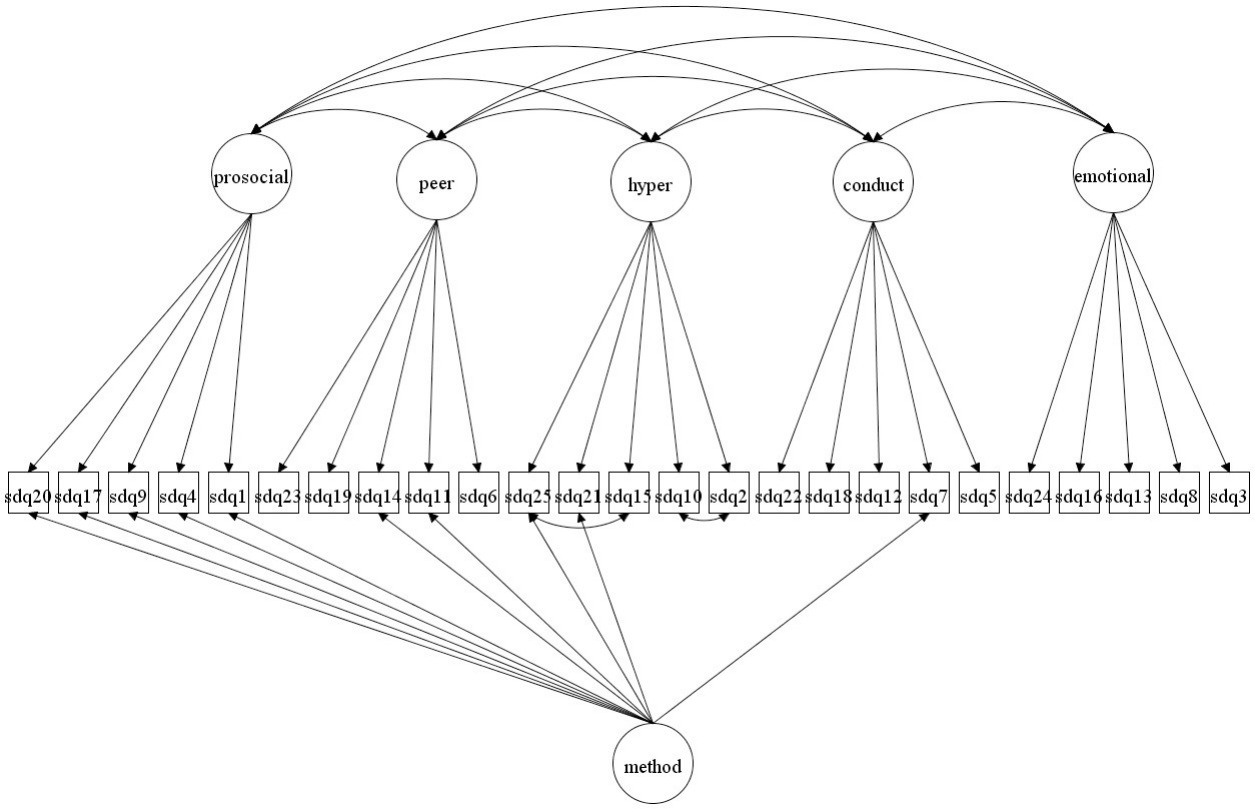
5-factor model with separate method factor and minor factors (Model 8b).

**Fig 16:**
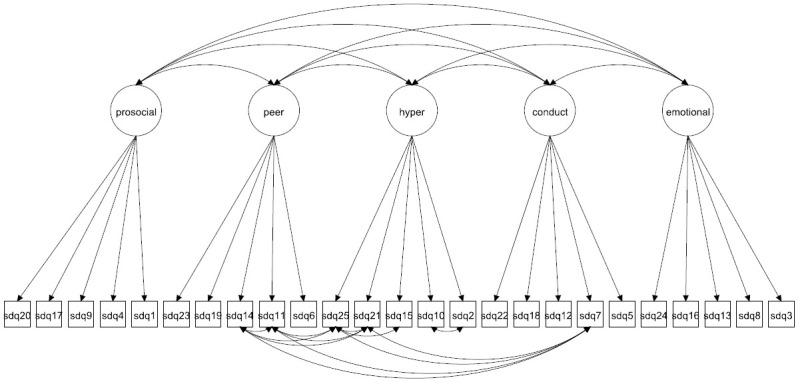
5-factor model with correlated errors for method effects and minor factors (Model 8c).

We focus on both the original 5-factor model and the 3-factor model as well as more complex 5- and 3-factor models allowing for method effects and minor factors. Given that the 3-factor model has been proposed to be more accurate in some studies [27, 37] it is crucial to compare how well this model fare compared with the 5-factor model. The models to be fitted are in Figs 1–16. Given our confirmative approach, we restrict our focus on minor factors to the hyperactivity scale. SDQ2 and SDQ10 can be expected to share residual variance because these two items both measure hyperactivity, whereas the other hyperactivity scale items measure inattention (SDQ15 & SDQ25) and impulsivity (SDQ21) [5, 25]. Consequently, SDQ15 and SDQ25 may be assumed to share error variance given that these two focus on inattention. Correlated error variances between these two sets of items were, for instance, specified in the study by Bøe et al. [35] and makes theoretical sense, but the potential of such minor factors need to be replicated.

## Methods

### Participants

The sample for this study consists of a nationally representative cohort of Swedish adolescents born in 2001 who take part in the ongoing Futura01-study. The sample is to be followed prospectively approximately every other year and our data concern the baseline measurements. The main focus of Futura01 is on the long-term consequences of substance use and abstinence in adolescence, but it also measures behavioural and emotional problems, sensation seeking and other psychological constructs.

The baseline data for Futura01 were collected in 2017 (between March and May) when participants attended 9^th^ grade (i.e., were 15–16 years). Data were collected in school during school-hours by means of a paper-and-pen questionnaire. Participants consenting to take part in the study were instructed to provide their personal identification numbers in the questionnaire. In all, it was possible to identify 5576 individuals with correct personal identity numbers, using the Luhn-algorithm. A small number of individuals (n = 27) were excluded because they failed to answer some central questions in the questionnaire or because of obviously false or exaggerated responses (e.g., reported that they had used each of a number of illicit substances 40 or more times), leaving a study sample of n = 5549 (50.5% females). There were low rates of missing values on the SDQ items, ranging from n = 19 (0.34%) for SDQ1 to n = 74 (1.33%) for SDQ14. Given the low prevalence of missing values, we did not consider imputing the missing values.

The Futura01 study has been approved by the regional ethics review board in Stockholm (2017/5:2).

### Measures

We used the Swedish translation of self-reported SDQ for individuals aged 11–17 years old, freely available at SDQ’s homepage [2]. As noted in the introduction, SDQ consists of 25 items of which 20 measure difficulties and five measure strengths. Table 1 shows the items together with the univariate responses. While the Swedish translation was used when collecting data, Table 1 uses the wordings in the English (UK) scale [2]. Each item in SDQ is assessed on a three-level scale where respondents choose among the options “Not true”, “Somewhat true” and “Certainly true”. The responses are assigned the values 0, 1 and 2. The responses may then be combined for each of the original five subscales, each scale thus ranging from 0 to 10. Totally five items on the difficulties scale are positively worded and need to be reverse coded before added to the sum total for each scale (SDQ7, SDQ11, SDQ14, SDQ21 and SDQ25, not reverse coded in Table 1). Presented in Table 1 is also the Cronbach’s alphas for the original five scales. Alpha values ranged from 0.52 for the Conduct problems scale to 0.76 for the Hyperactivity scale.

**Table 1:** Item responses for Strength and Difficulties Questionnaire (SDQ). Percent.

Item no		Not true	Somewhat true	Certainly true
	**Emotional problems (α = 0.70)**			
3	I get a lot of headaches, stomach-aches or sickness	54.3	32.7	13.0
8	I worry a lot	33.9	41.6	24.5
13	I am often unhappy, down-hearted or tearful	67.0	24.4	8.6
16	I am nervous in new situations. I easily lose confidence	26.8	46.1	27.1
24	I have many fears, I am easily scared	64.1	27.1	8.8
	**Conduct problems (α = 0.52)**			
5	I get very angry and often lose my temper	50.3	37.8	11.9
7	I usually do as I am told^a^	6.0	51.9	42.1
12	I fight a lot. I can make other people do what I want	89.8	8.6	1.6
18	I am often accused of lying or cheating	77.7	16.8	5.5
22	I take things that are not mine from home, school or elsewhere	86.5	10.9	2.6
	**Hyperactivity (α = 0.76)**			
2	I am restless, I cannot stay still for long^b^	30.6	52.4	17.0
10	I am constantly fidgeting or squirming^b^	36.4	43.2	20.4
15	I am easily distracted, I find it difficult to concentrate^c^	35.0	44.4	20.7
21	I think before I do things^a^	7.3	54.0	38.7
25	I finish the work I’m doing. My attention is good^a^^,^^c^	10.8	50.2	39.1
	**Peer problems (α = 0.55)**			
6	I am usually on my own. I generally play alone or keep to myself	45.8	41.7	12.5
11	I have one good friend or more^a^	1.4	6.6	92.0
14	Other people my age generally like me	5.2	40.9	53.8
19	Other children or young people pick on me or bully me	86.8	11.1	2.2
23	I get on better with adults than with people my age	40.6	47.1	12.3
	**Prosocial (α = 0.65)**			
1	I try to be nice to other people. I care about their feelings	1.5	23.7	74.8
4	I usually share with others (food, games, pens etc)	5.5	46.1	48.5
9	I am helpful if someone is hurt, upset or feeling ill	3.5	37.6	58.9
17	I am kind to younger children	3.2	19.1	77.6
20	I often volunteer to help others (parents, teachers, children)	4.5	48.9	46.6

^a^Positively worded difficulties item

^b^These items are hypothesized to constitute a minor factor in some of the CFA models tested

^c^These items are hypothesized to constitute a minor factor in some of the CFA models tested

### Statistical analysis

A series of factor structures of SDQ were tested using confirmatory factor analysis (CFA) (see Figs 1 to 16). CFA is known to be more appropriate than exploratory factor analysis (EFA) when testing a priori hypotheses. CFA is, similar to EFA, based on the common factor model but is more parsimonious [30]. In contrast to EFA where items are free to load on all factors in the solution, in CFA the researcher have to specify which factor(s) the items are to load on [30]. To account of the ordinal nature of the data, robust weighted least squares (WLSMV) models were fitted to a polychoric correlation matrix using Mplus vers. 8.5 [38]. Mplus by default uses all information available when there is missing data instead of removing individuals with missing data on any of the included indicators (i.e., listwise deletion). In the case of WLSMV this means using pairwise deletion [38]. One individual had missing data on all SDQ indicators and were thus excluded from analysis. There were non-missing data on 97.9% to 99.4% of the different pairs of SDQ items.

A total of 16 models were run. Model fit was judged primarily by the root mean square error of approximation (RMSEA), the standardized root mean squared residual (SRMR), the comparative fit index (CFI) and the Tucker-Lewis index (TLI). Influential recommendations are provided by Hu and Bentler [39] who suggest that for good model fit RMSEA should be about 0.06 or lower, that SRMR should be lower than 0.08, and that the CFI and the TLI should be about 0.95 or higher. However, other researchers recommend somewhat less strict cut-offs [30]. van de Looij-Jansen and colleagues [33], for instance, considered CFIs and TLIs in the neighborhood of 0.90 and RMSEA lower than 0.08 as “acceptable” in their study on the factor structure of SDQ in Dutch adolescents. We follow their approach here.

The χ2 value and the corresponding *p*-value are also presented for each model. If this is statistically significant, this suggests that the model does not provide a perfect fit to the observed data [40]. However, we do not rely heavily on this information as the χ2 test is considered too “stringent” and as it is almost always statistically significant in large samples [40].

As noted above, it is common in applied research to improve subsequent models by making modifications according to MIs, but this may lead to ad hoc modelling that makes little theoretical sense [36]. We consulted the MIs not to improve the models, but as an additional piece of information when assessing model fit. In brief, MIs are estimates of how much the χ2 value would decrease if allowing for the suggested modifications (e.g. allowing for covariation between the error variances for two items). MIs can be provided for all fixed or constrained parameters and they show the approximate change in χ2 when freeing a given parameter [30]. They thus compare χ2 in the constrained model with χ2 in a model in where one parameter is freely estimated and this difference is thus expressed as χ2 with 1 degree of freedom [30, 40]. A value of 3.84 or higher suggest that the differences in χ2 is statistically significant at p<0.05 (i.e., that the suggested modification would improve model fit significantly), but as also this test is affected by sample size it is recommended that also the “expected parameter change” values (EPCs) should be consulted [30]. Thus, we assessed the fully standardized EPCs which “provides an estimate of how much the parameter is expected to change in a positive or negative direction if they were freely estimated in a subsequent analysis” [30, p. 122]. EPCs may, for example, estimate how a given item would load on a factor that it was not specified to load on in the original model (e.g., if a hyperactivity item was free to load on the peer problem factor in SDQ).

We also checked the appropriateness of the different solutions. The model implied correlations between factors were inspected for impossible values (e.g., correlations exceeding 1.0) and we also checked the presence of any negative residual variances (i.e., an impossible value). The presence of impossible values indicates an improper solution [41]. In the results section, we note for which models such problems were encountered.

## Results

Table 2 presents fit indices for the CFA models. Models 1-4c concern the 3 factor-model whereas models 5-8c concern the 5-factor model.

**Table 2:** Goodness of fit indices for different factor structures of the Strength and Difficulties Questionnaire (SDQ).

Model		χ2	df	RMSEA (90% CI)	CFI	TLI	SRMR
1	3 factor model	8921.660***	272	0.076 (0.074, 0.077)	0.796	0.775	0.093
2a	3 factor model with cross-loadings^a^	7038.436***	267	0.068 (0.066, 0.069)	0.840	0.820	0.082
2b	3 factor model with separate method factor	Improper solution					
2c	3 factor model with correlated uniqueness	8605.252***	262	0.076 (0.074, 0.077)	0.803	0.774	0.091
3	3 factor model with minor factors	7481.517***	270	0.069 (0.068, 0.071)	0.830	0.811	0.087
4a	3 factor model with cross-loadings^a^ and minor factors	5691.011***	265	0.061 (0.059, 0.062)	0.872	0.855	0.076
4b	3 factor model with separate method factor and minor factors	Improper solution					
4c	3 factor model with correlated uniqueness and minor factors	7209.109***	260	0.069 (0.068, 0.071)	0.836	0.810	0.085
5	Original 5 factor model	6704.276***	265	0.066 (0.065, 0.068)	0.848	0.828	0.074
6a	5 factor model with cross-loadings^a^	5234.782***	260	0.059 (0.057, 0.060)	0.882	0.864	0.066
6b	5factor model with separate method factor	Improper solution					
6c	5 factor model with correlated uniqueness	6407.240***	255	0.066 (0.065, 0.067)	0.855	0.829	0.073
7	5factor model with minor factors	5635.596***	263	0.061 (0.059, 0.062)	0.873	0.855	0.071
**8a**	**5 factor model with cross-loadings and minor factors**	**4345.210** ***	**258**	**0.053 (0.052, 0.055)**	**0.903**	**0.888**	**0.063**
8b	5 factor model with separate method factor and minor factors	Improper solution					
8c	5 factor model with correlated uniqueness and minor factors	5423.836***	253	0.061 (0.059, 0.062)	0.878	0.855	0.069

***p<0.001

^a^Cross-loadings for the positively worded items sdq7 sdq11 sdq14 sdq21 and sdq25 on the prosocial scale

Note: df = degrees of freedom; RMSEA = Root mean square error of approximation; CFI: Comparative fit index; TLI = Tucker-Lewis Index; SRMR = Standardized root mean square residual. Best fitting model (8a) is in bold typeface.

The 3-factor model (model 1) showed quite a poor fit to the data. Although the RMSEA was acceptable, CFI and TLI were below recommended cut-offs. When allowing for cross-loadings of the positively worded difficulties items on the prosocial factor (model 2a), the fit improved but was still inferior (CFI = 0.84, TLI = 0.82). Model 2b, which included a separate methods factor, provided an improper solution. The correlated uniqueness model of the 3-factor solution (model 2c) had a poorer fit than model 2a, suggesting that a broader conceptualization of method effects including all positively worded items is preferable. However, also this model failed to provide a good fit to the data. Model 3 incorporated minor factors concerning the hyperactivity scale, whereas models 4a-4c in addition to minor factors also specified method effects (cross-loadings on the prosocial scale, a separate method factor and correlated error terms) in addition to minor factors. Similar to model 2b, the model incorporating a separate method factor (model 4b) provided an improper solution. Model 4a had the best fit to the data of all the models based on the 3-factor model of SDQ. While the CFI and TLI did not quite reach the level as would be required for an acceptable fit in the best model (i.e., model 4b), it is obvious that the fit of the 3 factor model improved when including method effects and minor factors.

The original 5-factor model did not fit the data particularly well, with CFI and TLI having lower value than generally recommended (0.85 and 0.83, respectively). With cross-loadings on the prosocial scale for the positively worded difficulties items, the fit improved but was still less than optimal. A similar pattern as for the 3-factor model emerged when comparing the three approaches to estimating method effects. The model with a separate method factor yielded an improper salutation. The model with cross-loadings of the positively worded difficulties items on the prosocial scale (model 6a) provided a relatively better fit than the correlated uniqueness model including CU’s for the positively worded difficulties items (model 6c), suggesting that a broader conceptualization of method effects fit the data better. However, neither model had a good fit to the data.

When adding minor factors to the original 5-factor model, there was a non-trivial improvement in model fit (model 7). The model also including cross-loadings of the positively worded items on the prosocial scale provided the best fit to the data, with the CFI exceeding 0.9 and the TLI approaching this level (model 8a). Also, the RMSEA was below the cut-off 0.06 and the SRMR was below 0.08 for this model. The 5-factor model with correlated uniqueness between positively worded difficulties items and minor factors (model 8c) was a clear improvement compared to the original 5-factor model, but its fit indices were inferior compared with model 8a.

Modification indices (MIs) suggested that there was room for improvement across models, with many MIs having high values and notable standardized expected parameter change (EPCs) values. We did not incorporate these to improve model fit, but it can be worth mentioning that, similar to Bøe et al. [35], a large MI and SEP value was found for a cross-loading of the “tantrum” item (SDQ5) on the emotional in the best fitting model (model 8a).

Table 3 shows the completely standardized factor loadings from model 8a, and the correlations between subscales from this model are in Table 4. As can be seen, the factor loadings for the positively worded difficulties items were larger on their primary scale than on the prosocial scale with the exception of SDQ7. Thus, while the fit of the 5-factor model was substantially improved when allowing for cross-loadings and minor factors, the original factor structure was broadly supported by the factor loadings. The correlations between SDQ2 and SDQ10 and between SDQ15 and SDQ25 were 0.59 and 0.27, respectively (not shown in Table 3).

**Table 3:** Completely standardized factor loadings from a 5-factor model with cross-loadings for positively worded difficulties items on the prosocial scale and minor factors (model 8a).

Item no		Emotional problems	Conduct problems	Hyperactivity	Peer problems	Prosocial
3	I get a lot of headaches, stomach-aches or sickness	0.58				
8	I worry a lot	0.71				
13	I am often unhappy, down-hearted or tearful	0.85				
16	I am nervous in new situations. I easily lose confidence	0.58				
24	I have many fears, I am easily scared	0.55				
5	I get very angry and often lose my temper		0.71			
7	I usually do as I am told		0.22			-0.34
12	I fight a lot. I can make other people do what I want		0.66			
18	I am often accused of lying or cheating		0.56			
22	I take things that are not mine from home, school or elsewhere		0.47			
2	I am restless, I cannot stay still for long			0.65		
10	I am constantly fidgeting or squirming			0.65		
15	I am easily distracted, I find it difficult to concentrate			0.79		
21	I think before I do things			0.39		-0.31
25	I finish the work I’m doing. My attention is good			0.68		-0.15
6	I am usually on my own. I generally play alone or keep to myself				0.58	
11	I have one good friend or more				0.71	-0.21
14	Other people my age generally like me				0.54	-0.33
19	Other children or young people pick on me or bully me				0.72	
23	I get on better with adults than with people my age				0.39	
1	I try to be nice to other people. I care about their feelings					0.77
4	I usually share with others (food, games, pens etc)					0.44
9	I am helpful if someone is hurt, upset or feeling ill					0.64
17	I am kind to younger children					0.64
20	I often volunteer to help others (parents, teachers, children)					0.68

All factor loadings are statistically significant at p<0.001

**Table 4:** Correlations between latent factors from model 8a.

	Emotional	Conduct	Hyperactivity	Peer problems	Prosocial
Emotional	1				
Conduct	0.39	1			
Hyperactivity	0.40	0.75	1		
Peer problems	0.62	0.32	0.16	1	
Prosocial	0.12	-0.45	-0.27	-0.09	1

All correlations are statistically significant at p<0.001

As shown in Table 4, the model implied correlations between subscales ranged from almost zero (prosocial and peer problems) to substantial. Strong correlations were found between peer problems and emotional (0.62) and between conduct and hyperactivity (0.75).

## Discussion

### Summary of findings

The Strength and Difficulties Questionnaire (SDQ) is one of the most popular screening instruments for emotional and behavioural problems in children and adolescents. This study used confirmatory factor analysis (CFA) to test its factor structure in a large and nationally representative study of Swedish adolescents in 9^th^ grade.

The original factor structure comprised of five correlated factors did not fit the data particularly well, and neither did the proposed 3-factor model comprised of three correlated factors. Although both of these models have been supported in prior exploratory factor analyses (EFA), the evidence from CFAs have been less conclusive. This is not surprising as CFA is a stricter approach than EFA [30]. A large number of models derived from prior studies were tested, incorporating both method effects and minor factors between items on the hyperactivity scale that theoretically could be expected to share variance above what can be accounted for by the latent hyperactivity variable. Different approaches to specify method effects in the SDQ were tested, entailing both cross-loadings of the positively worded difficulties items on the prosocial scale, a separate method factor as well as correlations between items presumed to drive the method effects (i.e., correlated uniquenesses). This was done for both the 3 and the 5-factor models.

Most models failed to provide a satisfactory fit to the data, as assessed by conventional criteria for goodness-of-fit [39]. Nonetheless, models differed on the fit indices and the 5-factor model incorporating cross-loadings for the positively worded difficulties items as well as minor factors had the best fit of the models tested. This model provided an acceptable, but not good fit. However, the factor loading of SDQ7 (“I usually do as I am told”) was higher on the prosocial scale than on the original scale (conduct problems), suggesting potential problems with using this item for assessing conduct problems by means of self-reports. Hagquist [14] has in a prior Swedish study on self-reported SDQ in adolescents pointed at problems with this item, showing that the conduct scale works better if this item is removed. Likewise, SDQ21 (“I think before I do things”) had quite a modest factor loading on its original scale (hyperactivity) and a non-trivial cross-loading on the prosocial scale. Similar findings based on self-reported SDQ have been noted in some prior research [22] and this item was found to be problematic in Hagquist’s Swedish study as well [14]. Van Roy and colleagues [22, p. 1308], in a Norwegian study, conclude that the meaning of SDQ7 and SDQ21 “may be unclear in self-report groups”.

Some prior studies have considered method effects by specifying an additional method factor including all 10 positively worded items in SDQ (e.g., [8]) but this provided an improper solution in this study. As a confirmatory approach was adopted here, we did not consider ways of overcoming the problems with improper solutions in these models. However, this may be an important task for future research. Nonetheless, the improvement in model fit when considering method effects is in accordance with findings from several prior CFAs of SDQ (see e.g., [3, 8, 22, 25, 28, 33]). Thus, while different approaches for accounting for method effects have been used, this and prior studies strongly suggest that method effects are crucial to consider when assessing SDQ’s factor structure.

The study also corroborates the importance of minor factor in SDQ [25, 28, 33, 35]. Models including correlated errors for two sets of items in the hyperactivity scale (for SDQ2 & SDQ10 and for SDQ15 & SDQ25) led to improvements in model fit, and this held true for both the 3 and 5-factor model. The role of minor factors in SDQ should be explored further, but for now there should be enough evidence to routinely include the two minor factors in the hyperactivity scale when analyzing SDQ’s factor structure [35]. The specification of these minor factors makes logical sense as the hyperactivity scale was developed explicitly to capture both hyperactivity and inattention (as well as impulsiveness) [5] but other minor factor may exist in SDQ. Several CFAs on SDQ have relied on modification indices (MIs) to guide the inclusion of CUs in the analyses, but a more conceptually driven specification of minor factors may be in order. It is generally recommended that the MIs should be used with caution (e.g., [30]) and it is worth remembering that the MIs that are obtained after running the analysis may be study-specific [36].

To make the research as cumulative as possible, it seems reasonable in future work to run replication studies of modifications done in prior studies. If specific minor factors are found to be present across several studies, research may then again move into explorative mode by checking the MIs. We believe that our understanding of SDQ’s factor structure will improve more by giving detailed consideration of a few minor factors at a time than to all possible modifications that could be done in any study to improve the fit. For example, in the current study many sizeable MIs were obtained after running the models, and the fit would have improved if the suggested modifications were made. However, several of the potential modifications did not make theoretical sense and such ad hoc changes may not work in other samples.

An important question regarding SDQ’s factor structure is whether cross-loadings should be allowed for. A typical CFA specifies that each of the included items should load on only one factor, as compared to EFA where each item loads on each factor. However, it has been argued that this model is “too restrictive” and that both theory and the items’ content often can support the inclusion of cross-loadings in factor structures [42]. Similar arguments have been put forth in a few recent studies on SDQ as well [43, 44]. For instance, if some or several items have non-trivial cross-loadings but their cross-loadings are constrained to zero, the correlations between factors may be inflated [42, 45].

In this study, the best fit was obtained in a model specifying cross-loadings of the positively worded difficulties items on the prosocial scale (as well as including minor factors). However, cross-loadings not primarily used to capture the effect of positive and negative wording may perhaps also be warranted. Bøe et al. [35], in a Norwegian study, included a cross-loading on the tantrum item (SDQ5) on the emotional scale based on modification-indices, and interpreted this as possibly due to the translation from SDQ from English to Norwegian. They point out that the wording in the Norwegian version of SDQ is similar to “irritability”, a criterion for both depression and anxiety in ICD-10 and DSM-IV [35]. Here, too, we found large modification indices for a cross-loading of the tantrum item (worded quite similar in the Swedish and Norwegian version) on the emotional problems scale so this issue may be explored further in subsequent work. Overall, we believe that more attention to translation issues may be needed. For instance, SDQ12 (“I fight a lot. I can make other people do what I want”) may have a different meaning in the Swedish translation. “Fight” is translated to “slåss eller bråkar mycket” in the Swedish version. “Slåss” means to hit someone, and thus has a narrower interpretation. This and some other items in SDQ also contain two clauses (e.g., SDQ1). This may make respondents uncertain what they “actually are supposed to respond to” [14, p. 1299). Future work should consider if fit may be improved by avoiding two clauses in the same item in self-assessed SDQ.

This study has addressed the factor structure of the entire SDQ but it should be noted that this is only one type of validation effort. Some researchers may be more interested in specific subscales. For instance, researchers specifically focusing on ADHD may be primarily interested in the hyperactivity subscale, whereas the conduct problems subscale may be of main interest for research on conduct problems. We emphasize that our results should not discourage researchers from using specific subscales in their research as the subscales agree at least moderately with clinical diagnoses [7]. In all, for the purposes of epidemiological research, SDQ seems to have acceptable factorial validity, provided that researchers acknowledge its weaknesses and specify method effects and minor factors.

While this study used a large, nationally representative sample of Swedish adolescents, it did not assess factorial validity of SDQ in clinical samples of Swedish adolescents. As SDQ is also indented for clinical assessment [2], studying the role of method effects and minor factors is crucial in clinical settings as well. However, there is a lack of studies on the factor structure of self-assessed SDQ in clinical contexts, and little corresponding work exists for parental reports as well [46]. Recently, Vugteveen et al. [46] showed that SDQ had a similar factor structure in both a community and a clinical sample using both self and parental reports. This suggests that “…SDQ screens for psychosocial problems in the same way in both settings” [46, p. 1487]. To the extent that a similar pattern holds in Sweden, our findings may inform clinical assessments as well. Future studies should explore whether Vugteveen et al’s findings extend to other countries, including Sweden.

### Strengths and limitations

A key-strength of this study is that it is based on a large, nationally representative sample of Swedish youth allowing us to make inferences to the national population of Swedish youth born in 2001. Several prior studies on SDQ’s factor structure have relied on more local data (e.g., from a given city or region) limiting their generalizability. The low levels of missing data on all the SDQ items is to be considered a further strength. Confirmatory instead of exploratory factor analysis was used to assess the factor structure of SDQ and this is considered more appropriate when testing a priori factor structures [30]. However, the use of CFA may potentially also be a limitation as CFA by some are considered too stringent [42]. As with all self-reported data, of course, we do not know how honest respondents were when answering the questions. There is also the potential that the Swedish version of the original SDQ may have lost some important nuances during translation and that this in part may explain the unsatisfactory fit of the original 5-factor model to the current data.

## Conclusion

This large scale study shows that the original 5-factor model of SDQ as well as the 3 factor model provided an inaccurate fit to data reported by a national sample of Swedish youth. Allowing for method effects and minor factors improved the fit of both the 5 factor and the 3 factor model but the 5 factor model had a better fit. The findings suggest that in epidemiological studies in youth SDQ should be conceptualized as comprising five rather than three factors.
